# Initial antimicrobial management of sepsis

**DOI:** 10.1186/s13054-021-03736-w

**Published:** 2021-08-26

**Authors:** Michael S. Niederman, Rebecca M. Baron, Lila Bouadma, Thierry Calandra, Nick Daneman, Jan DeWaele, Marin H. Kollef, Jeffrey Lipman, Girish B. Nair

**Affiliations:** 1 Pulmonary and Critical Care Medicine, New York Presbyterian/Weill Cornell Medical Center, 425 East 61st St, New York, NY 10065 USA; 2 Harvard Medical School; Division of Pulmonary and Critical Care Medicine, Brigham and Women’s Hospital, Boston, MA 02115 USA; 3 AP-HP, Bichat Claude Bernard, Medical and Infectious Diseas ICU, University of Paris, Paris, France; 4 Infectious Diseases Service, Department of Medicine, Lusanne University Hospital, University of Lusanne, Lusanne, Switzerland; 5 Division of Infectious Diseases, Sunnybrook Health Sciences Centre, University of Toronto, Toronto, Canada; 6 Department of Critical Care Medicine, Surgical Intensive Care Unit, Ghent University, Ghent, Belgium; 7 Division of Pulmonary and Critical Care Medicine, Washington University School of Medicine, St. Louis, MO USA; 8 Royal Brisbane and Women’s Hospital and Jamieson Trauma Institute, The University of Queensland, Brisbane, Australia; 9 Nimes University Hospital, University of Montpelier, Nimes, France; 10 Oakland University William Beaumont School of Medicine, Royal Oak, MI USA

**Keywords:** Sepsis, Antibiotic therapy, Antimicrobial therapy, Fungal infection, Pneumonia, Intra-abdominal infection, Pharmacokinetics, Bacteremia, Biomarkers

## Abstract

Sepsis is a common consequence of infection, associated with a mortality rate > 25%. Although community-acquired sepsis is more common, hospital-acquired infection is more lethal. The most common site of infection is the lung, followed by abdominal infection, catheter-associated blood steam infection and urinary tract infection. Gram-negative sepsis is more common than gram-positive infection, but sepsis can also be due to fungal and viral pathogens. To reduce mortality, it is necessary to give immediate, empiric, broad-spectrum therapy to those with severe sepsis and/or shock, but this approach can drive antimicrobial overuse and resistance and should be accompanied by a commitment to de-escalation and antimicrobial stewardship. Biomarkers such a procalcitonin can provide decision support for antibiotic use, and may identify patients with a low likelihood of infection, and in some settings, can guide duration of antibiotic therapy. Sepsis can involve drug-resistant pathogens, and this often necessitates consideration of newer antimicrobial agents.

## Background

Sepsis is a common and life-threatening illness in the ICU, requiring timely and effective antimicrobial therapy. The aims of this review are to identify the most common sites of sepsis, the likely pathogens, and the optimal approach to antimicrobial therapy. Effective therapy must be balanced by the need to avoid overuse of broad spectrum agents and thus must be accompanied by a commitment to antimicrobial stewardship. Using experts in this topic, we reviewed the literature relevant to antimicrobial management of sepsis and recommend key principles for management.

## Sepsis epidemiology, infection site and pathogens

Sepsis is a life-threatening organ dysfunction syndrome caused by a dysregulated host response to infection, associated with a mortality rate over 25%, that has been designated a global health priority [1–3]. The majority of sepsis is community-acquired, and progression can be insidious, making diagnosis difficult [3, 4]. Prognosis depends on early administration of broad-spectrum antibiotics and effective source control [5, 6].

Sepsis affects 1.7 million adults in the USA annually, with nearly 270,000 deaths [7], and between 19.4 and 31.5 million episodes annually, worldwide, with 5.3 million deaths [8]. A global study reported a decrease of 18.8% in sepsis incidence worldwide from 60 million cases in 1990 to 49 million cases in 2017 [9]. However, sepsis-related Medicare hospital admissions increased from 811,644 to 1,136,889 from 2012 to 2018, with an associated increase in hospital and subsequent skilled nursing care cost from $27.7 to $41.5 billion [10]. Mortality at 6 months remains high for septic shock at 60% and severe sepsis at 36% [10].

Bacterial infections are the most common cause, but viruses and fungi may occur in patients with comorbid conditions and immunosuppression. The most common foci in hospitalized patients are infections of the lower respiratory tract, followed by intra-abdominal, bloodstream, intravascular line infections, and urinary tract infections [11]. Major bloodstream isolates include *S. aureus, E. coli, Klebsiella* spp., *Pseudomonas aeruginosa*, Enterococci, Streptococci and coagulase-negative staphylococci [12]. In the Extended Prevalence of Infection in Intensive Care (EPIC III) study including 15,000 ICU patients from 88 countries, 65% of patients had at least 1 positive microbiological culture with gram-negative pathogens being most common (67%, *n* = 3540), including *Klebsiella* species, *E. coli, Pseudomonas* species, Enterobacteraceae, *Proteus, Stenotrophomonas, Serratia* and *Acinetobacter* species. Of the gram-positive microorganisms (37%, *n* = 1946)—S*. aureus, S. pneumoniae*, and Enterococcus were most common, and *Candida* species and *Aspergillus* were the common fungal microorganisms (16%, *n* = 864) [13]. Infection with specific multidrug resistant pathogens in the ICU [vancomycin-resistant Enterococcus(OR = 2.41), *Klebsiella* resistant to β-lactam antibiotics (OR = 1.29), carbapenem-resistant *Acinetobacter* species (OR = 1.40)] was independently associated with a higher risk of mortality compared to infection with other microorganisms [13].

In a study of 1072 patients with mostly community-onset sepsis, 61% had some health care exposure, including recent antibiotics, chemotherapy, wound care, dialysis, or surgery in the 30 days before sepsis onset, with a pathogen defined in 57% [4]. There was an increased 30-day mortality in those with underlying co-morbid conditions such as cirrhosis (OR = 3.59), immunosuppression (OR = 2.52), vascular disease (OR = 1.54) [4]. In another study including 2.2 million hospitalizations, Rhee and colleagues reported community-onset sepsis to be more common (87.9%, *n* = 83,620) than hospital-onset sepsis (12.1%, *n* = 11,534), but with a higher mortality in hospital-onset sepsis (OR = 2.1; 95% CI, 2.0–2.2) [14]. In a meta-analysis of 51 studies from both developing and developed countries, including neonatal ICUs, mortality was 52.3% (95% CI: 43.4–61.1%) in those with hospital-acquired sepsis [15]. Worldwide, age-standardized sepsis-related mortality is higher among males than females (164.2 vs.134.1 per100,000) and diarrheal illness and lower respiratory tract infections ranked 1 and 2 among the most common cause of sepsis-related mortality [9].

## The importance of early appropriate and timely therapy

Timely administration of appropriate antibiotic therapy (i.e., with activity in vitro against the causative pathogens) is the cornerstone of the management of serious ICU infections [1]. Observational, prospective and retrospective studies support the use of appropriate empiric antibiotic therapy in sepsis and septic shock [16–19]. Administration of inappropriate initial antibiotic therapy has been associated with greater mortality dating back to a prospective study in 1999, evaluating 2000 ICU patients [20]. These findings have been confirmed in a meta-analysis demonstrating reduced mortality (OR 0.44, 95% CI 0.38–0.50), and significantly shorter hospital lengths of stay, with corresponding reductions in hospital costs, in patients receiving early appropriate versus inappropriate antibiotic therapy in severe bacterial infection [21]. Similar associations for antifungal therapy in *Candida* bloodstream infections have been shown [22–24].

A retrospective cohort study of 21,608 adults with bloodstream infections from 131 US hospitals found that 4165 (19%) received discordant empiric antibiotic therapy (based on in vitro testing of blood culture isolates), which was independently associated with increased mortality risk (adjusted odds ratio 1.46 [95% CI, 1.28–1.66]) [25]. A retrospective cohort analysis of bloodstream infection with severe sepsis and septic shock found that the number needed to treat (NNT) with appropriate initial antimicrobial therapy to prevent one patient death was 4.0 (95% CI, 3.7–4.3) [26]. The prevalence-adjusted pathogen-specific NNT for appropriate therapy to prevent one death was lowest for multidrug-resistant (MDR) bacteria (NNT = 20), and higher for *Candida* spp. (NNT = 34), methicillin-resistant *Staphylococcus aureus* (MRSA; NNT = 38), and *Pseudomonas aeruginosa* (NNT = 38) [26].

The randomized prospective MERINO trial, that compared therapy with piperacillin-tazobactam to meropenem in patients with severe bloodstream infection caused by ceftriaxone-nonsusceptible *E coli* or *K pneumonia*e, supported early appropriate therapy [27]. Non-inferiority of the piperacillin-tazobactam arm could not be established with 23 of 187 patients (12.3%) randomized to piperacillin-tazobactam dying at 30 days compared with 7 of 191 patients (3.7%) randomized to meropenem (risk difference, 8.6%) [27].

**Fig. 1:**
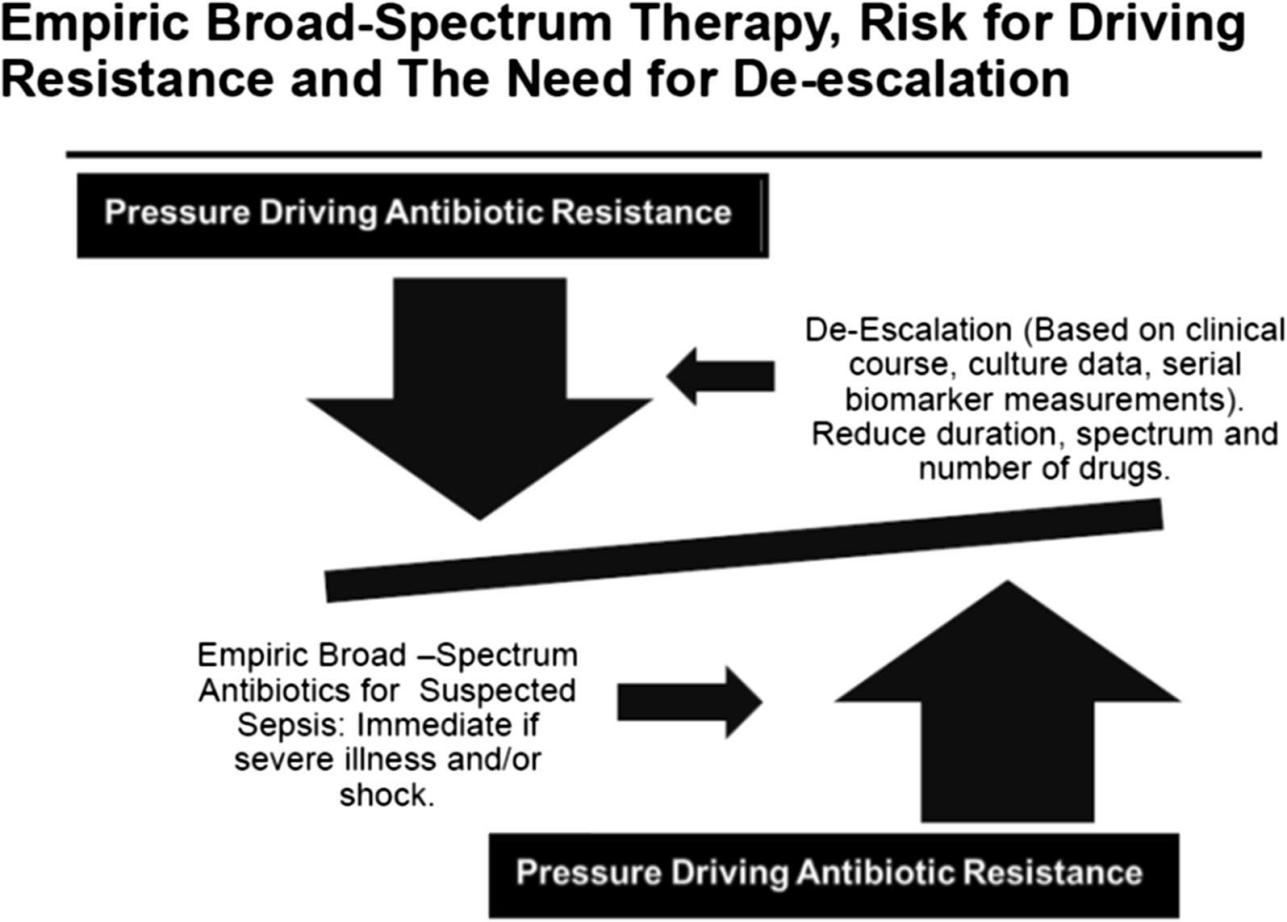
The need for immediate broad-spectrum empiric antimicrobial therapy for selected patients with severe sepsis may be life-saving, but may also put pressure to overuse antibiotics and drive antibiotic resistance. Thus, this approach comes with the obligation to try to control resistance by de-escalating therapy once serial clinical, microbiologic and laboratory data become available. De-escalation can be in the form of shorter duration of therapy, less broad-spectrum agents, fewer drugs, or a combination of these interventions

Delayed administration of appropriate therapy can be due to both delays in recognition of infection and administration of the antibiotics, but the optimal timing of therapy depends on the population studied [28]. A recent review suggested that a reasonable timeframe would be no later than three to five hours after infection onset, but immediately for patients with septic shock [29]. The administration of early appropriate therapy must be balanced against the unnecessary use of antibiotics, especially broad-spectrum agents, in the absence of proven infection, with excess mortality associated with this practice, and an increased risk of colonization and infection with antibiotic-resistant pathogens [30–33]. Thus, the use of rapid, broad-spectrum empiric therapy, especially in emergency settings, must come with a commitment to de-escalation, meaning shorter duration, less broad-spectrum therapy and fewer drugs, once clinical and microbiologic data become available (Fig. 1).

## Biomarkers to guide sepsis therapy

Clinical and biological signs of sepsis are neither sensitive nor specific, particularly in older patients and the immunocompromised, making decisions about starting and stopping antibiotics challenging in ICU patients [5]. Intensivists have sought biological markers to define when to safely postpone antibiotic therapy in those with possible infection. In 1998, a biomarker was defined as “a characteristic that is objectively measured and evaluated as an indicator of normal biological processes, pathogenic processes, or pharmacologic responses to a therapeutic intervention” [34]. Ideally, a sepsis biomarker should differentiate true sepsis from other inflammatory conditions in a timely and cost-effective manner, and be able to monitor the response to treatment, guiding both when to start and safely stop therapy. Sepsis activates multiple biochemical and immunological pathways, and the release of various molecules that could potentially serve as biomarkers [35]. Numerous promising biomarkers have been evaluated, but C-reactive protein (CRP) and procalcitonin are the most widely evaluated, in randomized controlled studies of antibiotic stewardship [35].

Given the heterogeneity and complexity of sepsis, no biomarker has sufficient accuracy to differentiate sepsis from other non-infectious causes of systemic inflammation, and biomarkers can only be used as adjuncts to clinical judgment, in defining when to start antibiotics [36]. However, combining information collected from several biomarkers could be valuable [37]. Development of molecular biology including “omics” could allow the development of better sepsis biomarkers [38], whose usefulness will be increased by their integration into biological scores [38]. Several host response gene-expression assays have been developed. SeptiCyteTM LB, (Immunexpress, Seattle, WA) has been cleared by the FDA in the United States, for discriminating sepsis from non-infectious systemic inflammation, among a heterogeneous cohort of 249 adult critical care patients [39].

Individualizing antibiotic treatment duration using biomarker guidance seems more intuitive than fixed duration in patients with sepsis. In a patient-level meta-analysis focusing on procalcitonin-guided antibiotic management (11 trials), using serial measurements, early discontinuation of antibiotics with a reduction in treatment duration was facilitated (9.3 days in 2252 procalcitonin guided patients vs. 10.4 days in 2230 control patients; *p* < 0.001), with significantly lower mortality with procalcitonin-guidance [40]. Importantly, CRP has been shown to be as useful as procalcitonin in reducing antibiotic use in a predominantly medical population of septic patients. Biomarkers decision support for sepsis management is more valuable to guide duration of therapy than to determine when to start antibiotic therapy. However, biomarkers with a high negative predictive value, along with clinical assessment, can help rule out infection and the need for immediate antibiotic therapy.

## Antibiotic therapy: principles of use and new agents

While appropriate therapy refers to the use of an antimicrobial agent to which the etiologic pathogen is sensitive, it is also necessary to administer the right dose, at the optimal time, that penetrates into the site of infection. This must be done without overuse that could drive antimicrobial resistance at a global level, as well as within the individual’s own bowel flora, which is often the source of ICU-acquired infections [41].

Optimal antibiotic usage involves avoidance of underdosing, while preventing adverse effects associated with overdosing. An initial large loading dose is required to “fill” the higher than usual volume of distribution in severe sepsis—roughly 1.5 times the standard dose [42]. Then dosing should occur according to drug clearance [42, 43]. Level 1 and 2 evidence suggests that double coverage for gram-negative infections is unnecessary [44, 45]. Still, some give one large initial dose of an aminoglycoside in ICU-infected patients, along with another agent, to assure broad enough coverage and to optimize rapid killing of the organism [42].

The kill characteristics of commonly used ICU antibiotics differ [43, 46]. For beta-lactams, the best effect is related to time above minimum inhibitory concentration (MIC) of the target pathogen; high daily doses are best administered by using continuous or extended infusions. While this could improve outcomes by keeping trough concentrations high especially in presence of resistance [47], not all data are supportive [48]. For aminoglycosides (a dose, or concentration-dependent antibiotic) therapy should be with large single daily doses (or extended interval in renal dysfunction) [49]. Quinolones, which also have dose-dependent killing, should also have higher, albeit spaced out, dosing.

Augmented renal clearance commonly occurs in younger patients without renal dysfunction [50] and necessitates higher than standard daily dosing to avoid subtherapeutic concentrations. With renal replacement therapy, underdosing and overdosing can occur, but higher doses of beta-lactams are probably a better option to prevent underdosing [46]. Therapeutic drug monitoring could be used as an aid to dosing most antibiotics [51]. When choosing antibiotics, the site of infection is important. Lipophilic antibiotics (e.g., quinolones) provide high concentrations in all tissues [49]. Hydrophilic antibiotics (eg aminoglycosides) do not penetrate well into tissues (lung, etc.) but stay in extravascular spaces, although beta-lactams penetrate better than aminoglycosides [49].

Previous antibiotic use predisposes patients to colonization with bacteria that are resistant to those drugs, and travel to areas with high prevalence of resistant organisms can lead to gut colonization with those endemic bacteria [52]. In addition, treatment in an ICU with high rates of local resistance can predispose to resistant pathogen infections.

A few new antibiotics can be used to treat severe infection due to resistant gram-positive and gram-negative bacteria [53, 54]. Ceftolozane-tazobactam is active against multidrug-resistant (MDR) *Pseudomonas aeruginosa*. Other newer agents, ceftazidime-avibactam, imipenem-relebactam, meropenem-vaborbactam, and cefiderocol can be used in patients with risk factors for resistant pathogens that are particularly susceptible to these agents. For those with carbapenem-resistant *Enterobacteriaceae*, ceftazidime-avibactam, imipenem-relebactam, and meropenem-vaborbactam may be most effective. For organisms that produce metallo-beta-lactamases ceftazidime-avibactam and cefiderocol would be good options [53, 54]. In the future, phage therapy may be a therapeutic option that needs study in sepsis [55].

## Pneumonia: initial empiric therapy for CAP, HAP, VAP

*Severe community acquired pneumonia (CAP)* [56–59]. For patients without risk factors for MRSA or *Pseudomonas aeruginosa* (PSA) infection, the currently recommended initial empiric therapy is (a) beta-lactam plus a macrolide or (b) beta-lactam plus a respiratory fluoroquinolone (FQ), both of which are acceptable, although more evidence favors a beta-lactam/macrolide. While evidence supporting these recommendations is based upon observational studies, a meta-analysis and systematic review found improved mortality for treatment with beta-lactam/macrolide over a beta -lactam/FQ, especially with severe CAP [60, 61]. There are not sufficient data to recommend treatment with FQ monotherapy or a beta-lactam plus doxycycline in severe CAP.

Patients with risk factors for MRSA or PSA might have been characterized as healthcare-associated pneumonia (HCAP) in the past, but his term has been abandoned [56]. Multiple studies demonstrated that HCAP risk factors did not necessarily predict the presence of resistant organisms and that coverage for these organisms did not improve clinical outcomes [62–64]. The 2019 ATS/IDSA guideline recommends empiric MRSA and/or PSA coverage for CAP patients with risk factors for these pathogens, followed by de-escalation of therapy, if cultures are negative. The best risk factors for MRSA and PSA infection are previous growth of these pathogens [65–67], as well as recent hospitalization and parenteral antibiotic exposure (within 90 days) [68–70]. The development of validated scoring systems that accurately predict risk for these pathogens has proven difficult, as have efforts to develop locally validated risk factors. Empiric MRSA and/or PSA coverage in severe CAP, with de-escalation, has proven to be a safe strategy [71–73]. However, a recent study found a low overall rate of de-escalation in the setting of negative cultures, providing an opportunity to improve antibiotic use [74]. Possible empiric regimens recommended for MRSA pneumonia include vancomycin or linezolid [75]. Therapy for PSA includes piperacillin/tazobactam, cefepime, ceftazidime, aztreonam, meropenem, or imipenem. Newer agents may also have a role.

*Hospital- and ventilator-acquired pneumonia (HAP, VAP)*. Local antibiograms are recommended to guide empiric antibiotic coverage [76]. All VAP patients should receive *S. aureus* and PSA/gram-negative coverage empirically, with additional consideration of resistant organisms in those with risk factors. These include prior antibiotic use within 90 days, septic shock or ARDS, at least 5 days of hospitalization in the past 90 days, and requirement of acute renal replacement therapy, although not all studies have validated these risk factors. MRSA coverage for VAP is recommended for patients with at least 1 of these risk factors and where local prevalence of MRSA is not known, or is > 10–20% of *S. aureus* isolates. Two anti-pseudomonal agents from different classes are recommended for VAP patients with at least 1 risk factor for resistant organisms and where the local prevalence of gram-negative resistance to a single anti-pseudomonal agent is not known, or is > 10% of gram-negative isolates. Treatment is identical in HAP as in VAP. Guidelines support empiric coverage for drug-resistant pathogens in at risk patients, with subsequent de-escalation if cultures are negative [76–79]. For all at-risk patients, the initial empiric regimen should include coverage for methicillin-sensitive *S. aureus* and PSA/gram-negatives (e.g., piperacillin/tazobactam, cefepime, imipenem, meropenem, ceftolozane/tazobactam). Recommended regimens for MRSA and resistant PSA are similar to those described above for severe CAP.

## Intra-abdominal infections

Complicated intra-abdominal infections (cIAI)—which refers to the extension of the disease process beyond the initial focus of infection, e.g., diffuse peritonitis after diverticulitis—are typically diagnosed before ICU admission but may also develop during ICU stay, often after surgery (80). cIAI is typically polymicrobial, with both aerobic and anaerobic bacteria. Among gram-negative pathogens, Enterobacterales are most common, and non-fermenting pathogens such as *Pseudomonas* or *Acinetobacter* spp. are not as frequent as in respiratory or bloodstream infections [13, 81]. Enterococci are particularly prevalent in critically ill patients with cIAI—they represent roughly half of the gram-positive isolates [13, 81]. However, anaerobic bacteria may be difficult to culture.

While sampling the source of infection is only done at a later stage during a procedure to control the source of infection (either percutaneous drainage or open surgical approach), empirical antimicrobial therapy should not be delayed. Blood cultures should be taken, but the relevance of sampling abdominal drains is limited. Empiric therapy should cover a wide spectrum of pathogens, e.g., a broad-spectrum beta-lactam/beta-lactamase inhibitor combination or a carbapenem, adapted to the local ecology. However, in the therapy of Enterococci, some strains may not be susceptible to beta-lactam antibiotics, particularly after recent exposure to drugs from this class, and empirical therapy with glycopeptides or oxazolidinones should be considered. When using empiric carbapenem treatment, it is essential to confirm enterococcus susceptibility.

Critically patients with cIAI often have multiple risk factors for invasive candidiasis, and empirical antifungal therapy is generally recommended for the most severely ill [82]. In a recent global study, fungi were involved in 13% of the patients, with *Candida albicans* isolated in two-thirds of those patients. Either azoles or echinocandins can be used empirically, based on the severity of illness, local epidemiology and previous exposure to antifungal drugs.

Controlling the source of the infection is essential, and should be pursued as soon as logistically possible [5, 80]. Percutaneous drainage is preferred if the infection is localized and no ongoing contamination of the abdomen is present.

## Empiric therapy for bacteremia

Among patients admitted with sepsis, nearly half remain culture-negative [83, 84]. However, among culture positive patients, microbiologic data from the bloodstream offers an important opportunity to modify therapy. In bacteremic infection, there are three empiric windows prior to definitive susceptibility results: (1) syndrome-guided therapy, (2) gram stain morphology-guided therapy, and (3) pathogen-guided therapy [85].

The hospital antibiogram, for each specific drug-bug combination, can aid in selecting empiric antibiotic treatments prior to the availability of susceptibility results, in pathogen-guided therapy (empiric window 3). Recently, this window has expanded to earlier time points, due to rapid pathogen identification methods such as matrix absorption laser desorption/ionization time of flight (MALDI-TOF), which have outstripped rapid susceptibility testing methods [85]. Prior to window 3, a weighted incidence hospital antibiogram can provide the overall susceptibility rates among all gram-negative bacteremias, that can be used to guide treatment in empiric window 2. Pulling together historic susceptibility information by syndrome is more challenging, but is worthwhile to inform local guidelines for empiric treatment of syndromes (window 1) such as central line associated bloodstream infection (CLBSI) [86], and intra-abdominal/hepatobiliary infections [87].

**Fig. 2:**
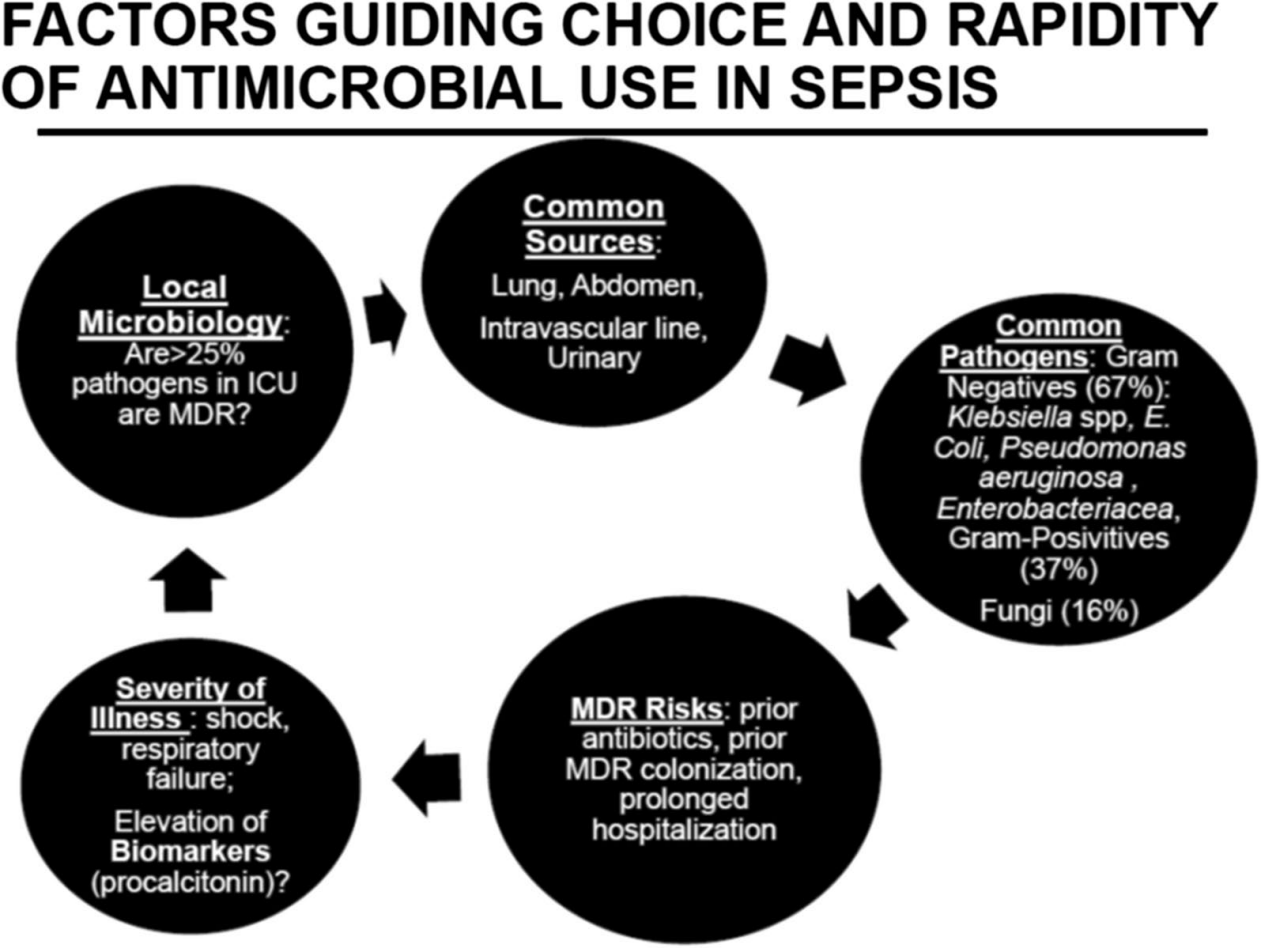
The rapidity of empiric therapy and the choice of specific agents are determined by the clinical scenario of the patient with suspected sepsis. Immediate therapy is given to those with a high likelihood of infection, and severe illness and or shock. If biomarkers like procalcitonin are not elevated, and the patient is not severely ill, immediate therapy is not necessary, and some patients may not even have infection. Specific agents are chosen with a consideration of the most common site of infection (lung > abdomen > catheter-associated infection > urinary tract infection). Each site has a group of likely pathogens, but these can vary, depending on patient-specific risk factors for resistance, and local ICU patterns of drug-resistant organisms. In sepsis, gram-negatives are more common than gram-positive, but some patients may also have fungal infection

To operationalize the antibiogram, we need to know what threshold of coverage to target with empiric treatment. In one physician survey, the median preferred thresholds for adequate coverage were 80% for mild sepsis and 90% for severe sepsis [88]. Using a 90% minimum threshold, for infections like VAP and CLABSI, we will potentially need to recommend toxic (e.g., aminoglycoside), reserved (e.g., carbapenem), or toxic and reserved (e.g., colistin) combinations of antibiotics for almost every patient, further driving antibiotic resistance. The solution is to use known predictors of antimicrobial resistance to individualize empiric antibiotic treatment so that we can use narrower spectrum monotherapy regimens when they will suffice, and limit broader spectrum combination therapy regimens to those who most need them (Fig. 2). Decision support models for empiric treatment of gram-negative bacteremia can incorporate risk factors for resistance (patient demographics, recent hospital exposure, recent antibiotic use, prior microbiology culture results) and promote rapid de-escalation of antibiotics without compromising time-to-adequate treatment [89, 90]. As discussed for pneumonia, patient’s prior microbiology results provide powerful information to predict resistance for current infections [91, 92]. With *Staphylococcus aureus* bacteremia, a prior positive MRSA surveillance swab result necessitates use of anti-MRSA empiric treatment [93]. With gram-negative bacteremia, identification of a prior gram-negative organism resistant to a specific drug within the last year should preclude use of that antibiotic [91].

## The approach to fungal sepsis

Invasive fungal infections (IFI) are rising as a cause of ICU sepsis and are associated with a mortality of 40% to 60% [94]. Epidemiological data, risk factors, prediction rules, scores, microbiological data and biomarkers can help identify patients with fungal sepsis.

Fungi account for around 5% of all cases of sepsis. *Candida,* the main etiological agent, is the sixth to tenth most common agent of bloodstream infections and often presents as candidemia or deep-seated candidiasis [95, 96]. One-third of all candidemia occurs in the ICU and 25% to 35% of candidemic patients present with sepsis or septic shock [95, 97]. Pneumocystosis, cryptococcosis, histoplasmosis, invasive aspergillosis, mucormycosis, fusariosis, penicilliosis and scedosporiosis may occasionally also present with disseminated infection and sepsis.

*Candida* is a normal constituent of the microbiota of the skin, the gastrointestinal tract, the urethra and the vagina, making it difficult to distinguish colonization from infection when isolated from non-sterile body sites. As an opportunistic pathogen, *Candida* is unlikely to cause infections without either a profound alteration of the microbiota, the integrity of the skin or the mucous membranes, or of host defenses [94, 98]. In immunocompetent ventilated ICU patients, isolation of *Candida* from lower respiratory tract specimens nearly always indicates colonization rather than infection [99]. A *Candida* colonization index of 0.5 or greater, defined as the ratio of positive to negative screening cultures of body sites, may increase the likelihood of invasive candidiasis, and may trigger preemptive or empiric therapy [100, 101].

Risk factors for invasive candidiasis are non-specific and similar to bacterial infections, (prior colonization, broad-spectrum antibiotic therapy, intravenous access devices, parenteral nutrition, diabetes, renal insufficiency, hemodialysis, abdominal surgery, pancreatitis, neutropenia, solid organ transplantation and immunosuppressive therapy) [94]. Scoring systems based on combinations of risk factors, underlying conditions and clinical characteristics exhibit high (> 90%) negative predictive values for IFI and may assist in ruling out invasive candidiasis [102–104].

Culture-based diagnostic tests for IFI are not sensitive and have a long turnaround time, resulting in delayed initiation of targeted antifungal therapy. Blood cultures are negative in 30% to 50% of candidemia patients and in 80% to 90% of patients with primary deep-seated candidiasis [105]. Sensitivities and specificities of non-culture based tests are in the range of 75% to 95% for mannan/anti-mannan antibody, β-D-glucan and polymerase chain reaction and 45% to 95% for *Candida albicans* germ tube assay [105–107]. The T2Candida panel looks promising in early clinical trials [108].

Given the high mortality associated with IFI, especially in immunocompromised patients, prompt initiation of pre-emptive or empirical antifungal therapy is critical [94, 98]. The clinical conditions (primary site of infection, immune status of the host), local epidemiology, microbiology and fungal biomarker data, prior exposure to antifungal agents and potential drug interactions will guide the choice of antifungal therapy. Echinocandins are the preferred agents for the treatment of invasive candidiasis [109–111]. However, the emergence of echinocandin-resistant *C. albicans, C. glabrata* and *C. auris* is a concern [112, 113]. Triazoles or lipid formulations of amphotericin B are the preferred agents for mold infections [114]. Step-down therapy will depend on the response to initial therapy and culture results. Appropriate source control (drainage of collections, removal of catheters or of prosthetic devices, whenever possible) is a critical component of management.

## Conclusions

The management of suspected sepsis requires thoughtful and individualized care. Initial empiric therapy should be immediate for those with a high likelihood of infection, severe illness and/or shock. Specific empiric antimicrobial therapy should be chosen with consideration of the likely site of infection, common pathogens for these sites, and with modification made by consideration of patient-specific risk factors for resistance and knowledge of local microbiology. While timely and appropriate therapy is necessary to reduce mortality, it must be accompanied by a commitment to de-escalate once we get culture and serial clinical and laboratory data, since indiscriminate use of broad-spectrum empiric therapy is a driving force for antimicrobial resistance. Key recommendations for management are summarized in Table 1.

**Table 1:** Summary and Key recommendations

1	Sepsis mandates *prompt antibiotic therapy and source control*
2	Bacteria are the most common cause of sepsis, but viruses and fungi can also be responsible. Gram-negative organisms are more common than gram-positives, but many bacteria are *multidrug resistant (MDR), which should be considered when choosing empiric therapy*
3	Use of *initial appropriate therapy* leads to reduced mortality, length of stay and cost, and *should be selected based on* the suspected source of infection, the likelihood of MDR pathogen infection, and consideration of local microbial susceptibility patterns. *Initial therapy should be no later than three to five hours after infection onset, but immediately for patients with septic shock, and for those with severe illness and a high* likelihood of infection
4	*Biomarkers* such as procalcitonin and C-reactive protein may have a role in antimicrobial stewardship, but *should not be used alone to determine whether to start antibiotic therapy in patients with sepsis*
5	Even with appropriate antibiotic therapy, it is *necessary to use the correct dose, often higher than usual in septic patients, who can have augmented renal clearance* of antibiotics, along with alterations in volume of distribution, cardiac output and penetration to the site of infection
6	Empiric therapy for septic patients with pneumonia (CAP, HAP, VAP) *should never be with a single agent*, and is based on risk factors for MDR pathogens, with *a focus on initially broad spectrum therapy, followed to de-escalation if MDR pathogens are not present on culture*. The most important risk factors to consider when choosing empiric therapy are *local microbiology, recent use of broad spectrum antibiotics in the past 90 days, recent hospitalization for at least 5 days in the past 90 days, and prior colonization or infection* by MRSA or *Pseudomonas aeruginosa*
7	*Complicated intra-abdominal infection (cIAI)* is often polymicrobial, involving gram-negatives, anaerobes and enterococci. *Initial empiric therapy of septic patients should be with a beta-lactam/beta-lactamase inhibitor or a carbapenem*, *and in some patients*, *Candida species should be targeted with added coverage*. Management also includes source control with percutaneous or surgical drainage, which can also obtain material for culture
8	Empiric therapy of bacteremia begins on a syndromic basis prior to the positive blood culture result, and then can be modified when gram stain and then pathogen identity are known. The latter window is becoming possible at earlier time points, due to the advent of rapid microbiologic testing. *Therapy choices should be based on individual patient risk factors for specific pathogens, local microbiology, and done with a goal of covering the etiologic pathogen at least 90% of the time*
9	Fungal infection accounts for 5% of sepsis, is most commonly due to *Candida* spp. and can be predicted by prediction scores, epidemiologic data, microbiologic data and biomarkers. Risk factors overlap with those for other causes of ICU sepsis. *Pre-emptive and empiric therapy are often necessary and echinocandins are preferred for Candida, but some strains are becoming resistant*

## Data Availability

Not applicable.
